# A new species of *Cales* (Hymenoptera: Aphelinidae) parasitizing *Bemisia pongamiae* (Takahashi) (Hemiptera: Aleyrodidae) in Taiwan, with a key to world species of the *Cales spenceri*-group

**DOI:** 10.3897/BDJ.3.e6352

**Published:** 2015-10-26

**Authors:** Andrew Polaszek, Yuan-Tung Shih, Samantha E. Ward

**Affiliations:** ‡ Natural History Museum, London, United Kingdom; § National Taiwan University, Taipei, Taiwan

**Keywords:** Parasitoid, taxonomy, whitefly pests

## Abstract

**Background:**

The genus *Cales* has been extensively revised recently and divided into two species groups, the *noacki*- and *spenceri*-groups Mottern et al. (2010), Mottern and Heraty (2014).

**New information:**

*Cales motterni* Polaszek, Shih & Ward **sp. nov.** is described from two females reared from the whitefly *Bemisia pongamiae* from Taiwan. The species belongs to the *spenceri-* group, and has a characteristic and unusual antennal clava. A key to the four species currently known from the *spenceri*-group is provided.

## Introduction

Species of *Cales* Howard 1907develop as primary parasitoids of whiteflies, with a record of *Cales noacki* Howard from eggs of Lepidoptera (Viggiani and Battaglia 1984). They are unusual in having the ability, at least in *C. noacki*, to parasitise 2nd-4th instars, and to emerge from 3rd or 4th instars (Miklasiewicz and Walker (1990). The developmental stages of *C. noacki* are very unusual among chalcidoids (Laudonia and Viggiani 1986), and presumably this applies to other members of the genus.

*Cales* has always been a unique and enigmatic genus, with placement in the Aphelinidae regularly questioned and discussed (Mottern et al. 2010), but recent combined analyses of morphology and DNA have suggested a close relationship with *Eretmocerus* (Heraty et al. 2013).

Mottern et al. (2010) included the following species in the *Cales spenceri* species-group: *C. spenceri* (Girault), *C. orchamoplati* Viggiani & Carver and *C. berryi* Mottern & Heraty (in Mottern *et al* 2010).

## Materials and methods

A series of surveys was undertaken from 2004 to 2014 for the collection of parasitoid host whiteflies, scale insects and aphids in Taiwan. Whiteflies were identified by C.C. Ko, National Taiwan University, where the holotype and paratype are deposited. Collection and rearing methods during this survey are detailed by Shih et al. (2015), based partly on those described in Noyes (1982).

### Terminology

Morphological terminology and the format for species descriptions follow Mottern et al. (2010). Photographs were made using a Leitz Ortholux compound microscope with Nomarski Differential Interference Contrast illumination. Images were processed using the stacking software Automontage (Synoptics, Cambridge, UK), and further edited with Adobe Photoshop CC 2014.

### Abbreviation

NTU: National Taiwan University

## Taxon treatments

### Cales motterni

Polaszek, Shih & Ward, 2015 sp. n.

urn:lsid:zoobank.org:act:81A93438-A9A8-45D3-9091-1EB842972CCA

#### Materials

- **Type status:** Holotype. **Occurrence:** recordedBy: Y.T. Shih; individualCount: 1; sex: female; previousIdentifications: ex *Bemisia pongamiae*; **Location:** country: TAIWAN; stateProvince: Xindian District; locality: Wulai; locationRemarks: on *Acer* sp.; **Event:** eventDate: 10.xii.2010; **Record Level:** type: on slide; institutionID: NTU
- **Type status:** Paratype. **Occurrence:** recordedBy: Y.T. Shih; individualCount: 1; sex: female; previousIdentifications: ex *Bemisia pongamiae*; **Location:** country: TAIWAN; stateProvince: Xindian District; locality: Wulai; locationRemarks: on *Acer* sp.; **Event:** eventDate: 10.xii.2010; **Record Level:** type: on slide; institutionID: NTU

#### Description

##### Female holotype (Fig. 1-8)

Colour: pale brown; vertex of head and ante­rior half of mesoscutum orange; posterior half of mesoscutum and scutellum brown; face and legs pale, almost white.

**Figure 1.**
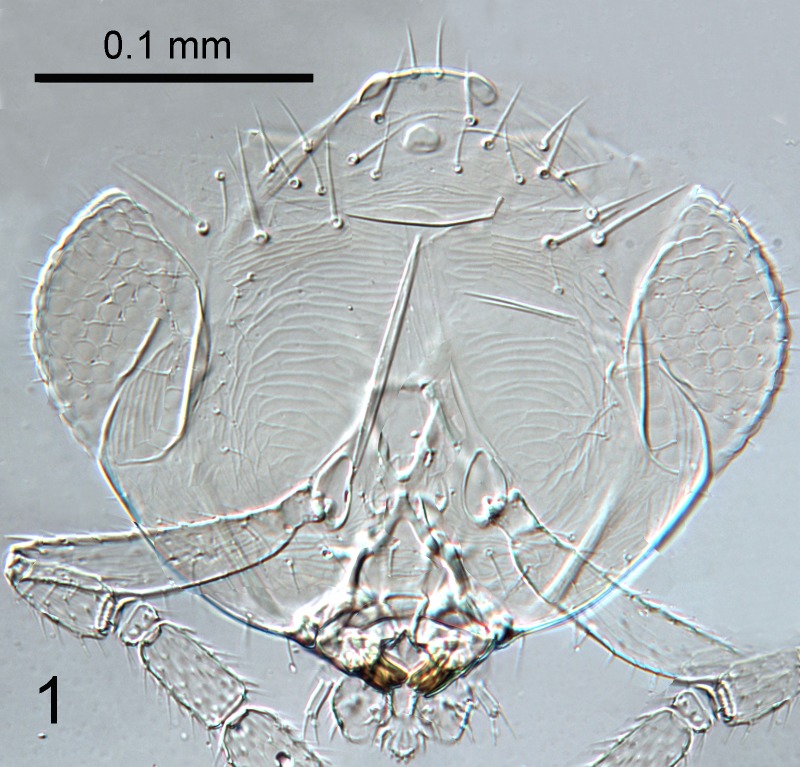
*Cales motterni*: face

**Figure 2.**
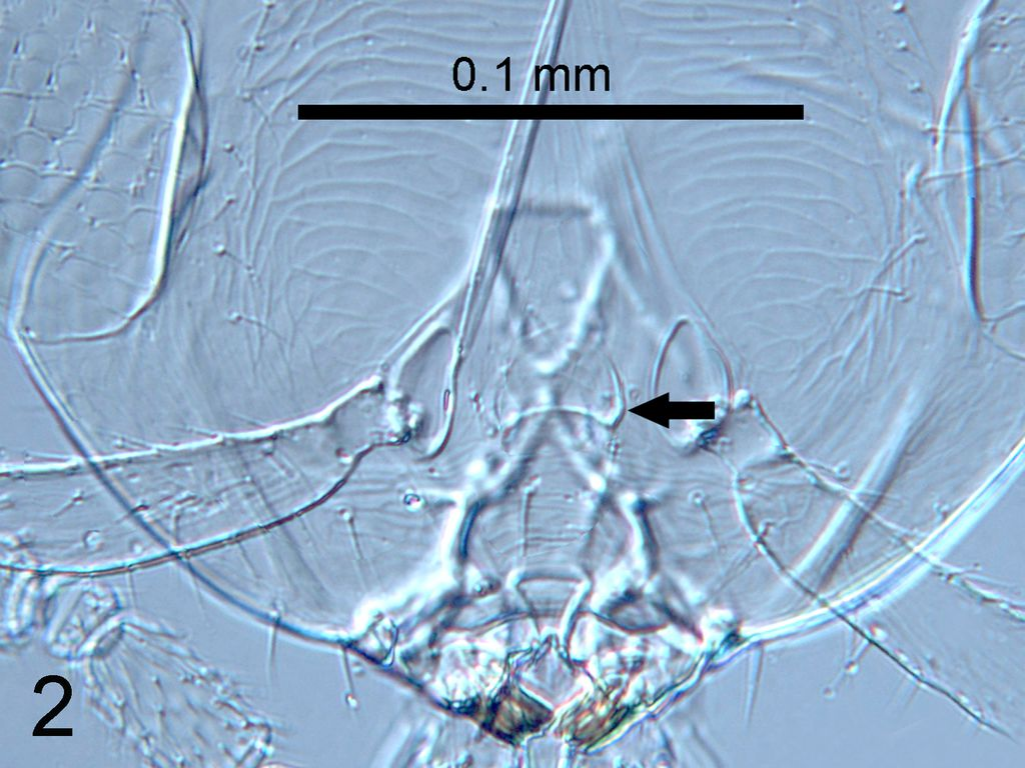
*Cales motterni*: detail of face; inter-antennal protuberance arrowed

**Figure 3.**
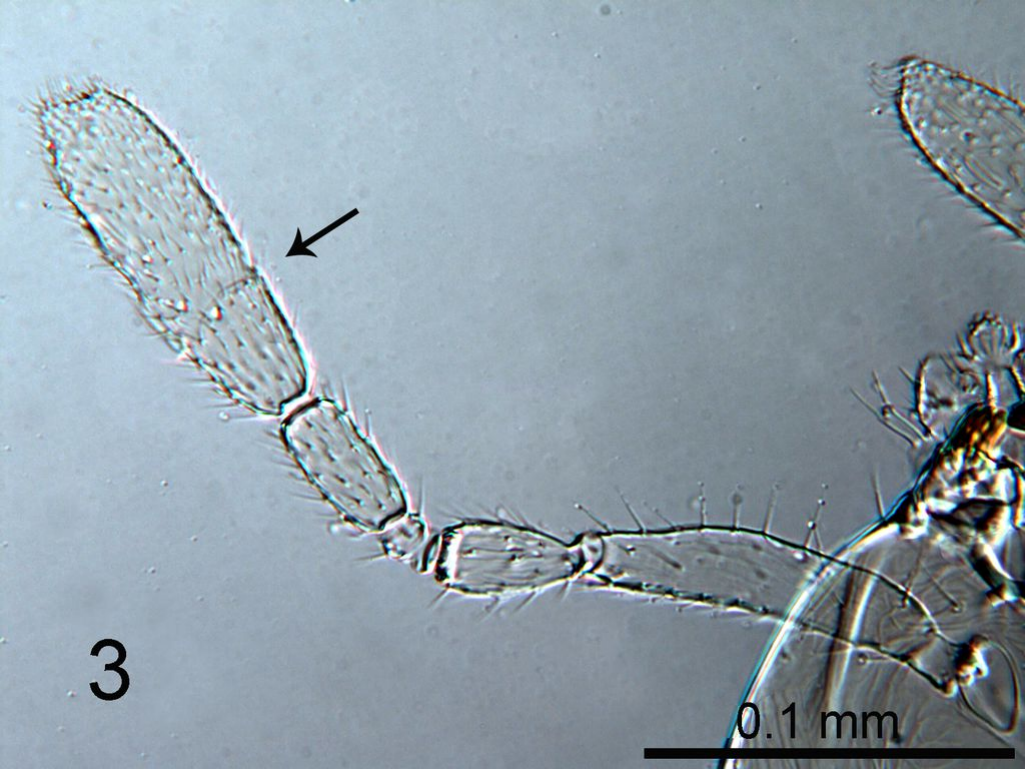
*Cales motterni*: antenna: clava suture arrowed

**Figure 4.**
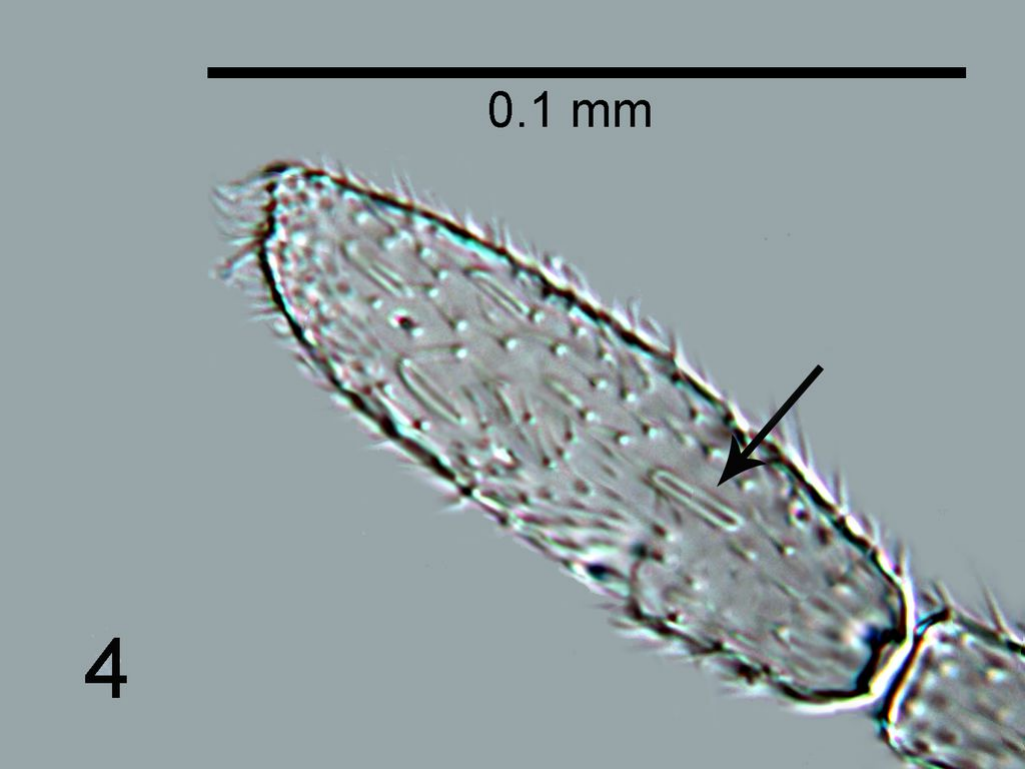
*Cales motterni*: antennal clava with multiporous plate sensillum arrowed

**Figure 5.**
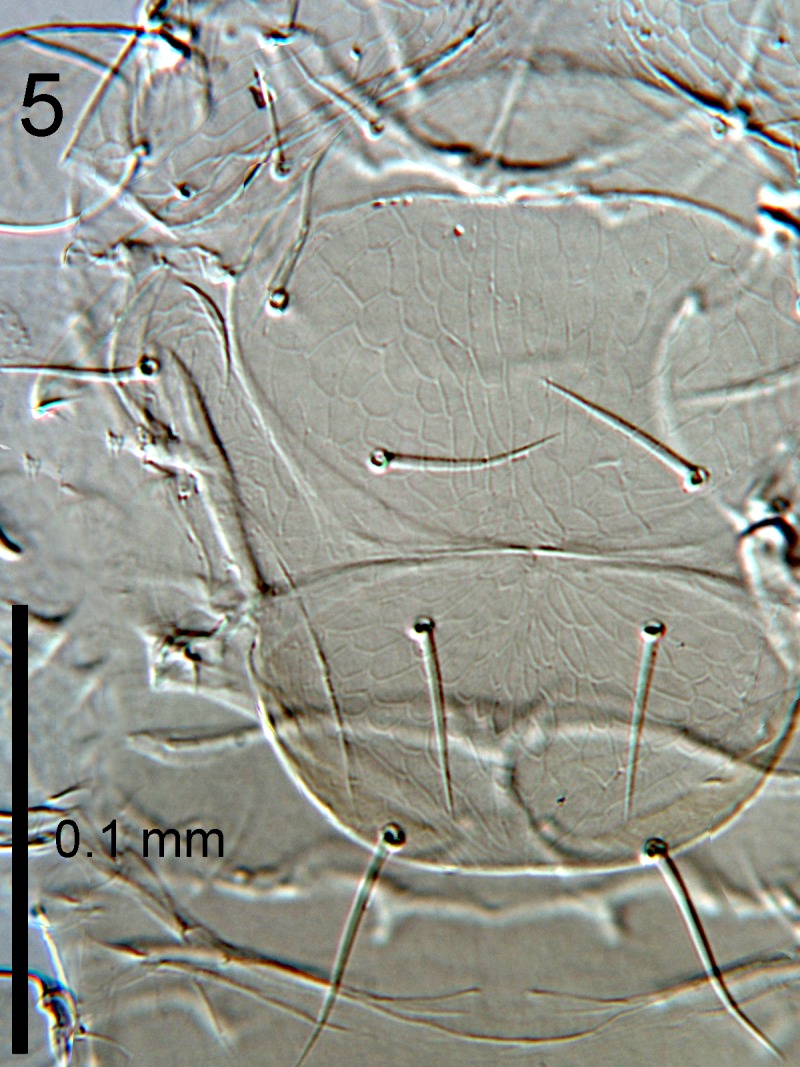
*Cales motterni*: mesoscutum and scutellum

**Figure 6.**
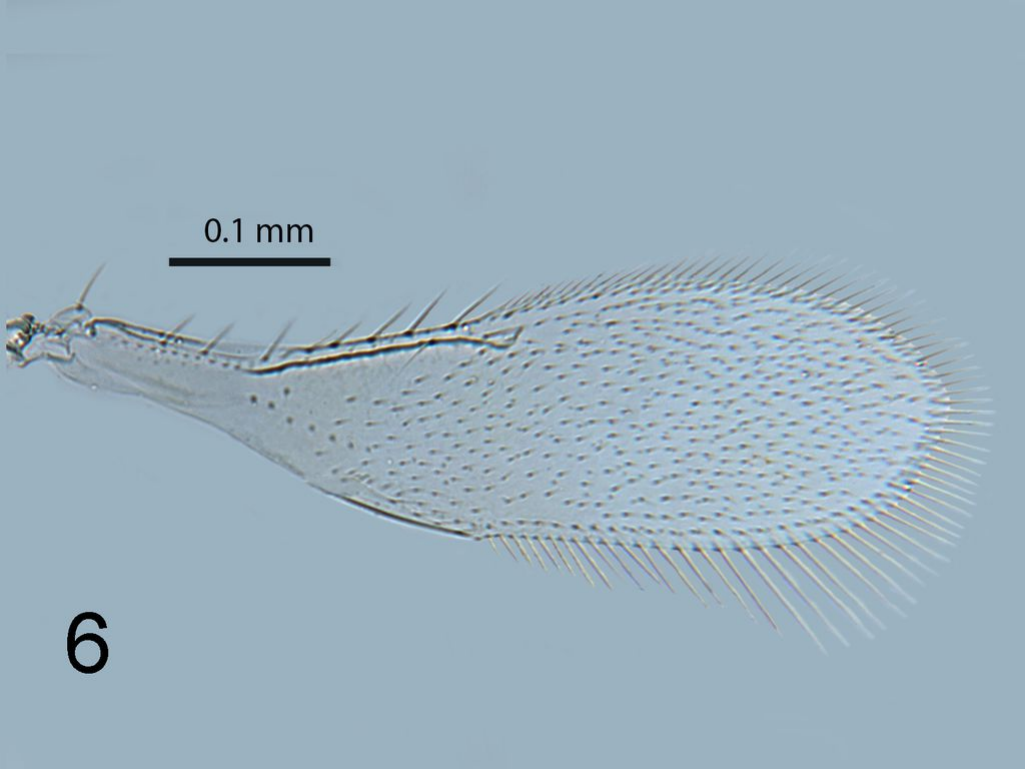
*Cales motterni*: fore wing

**Figure 7.**
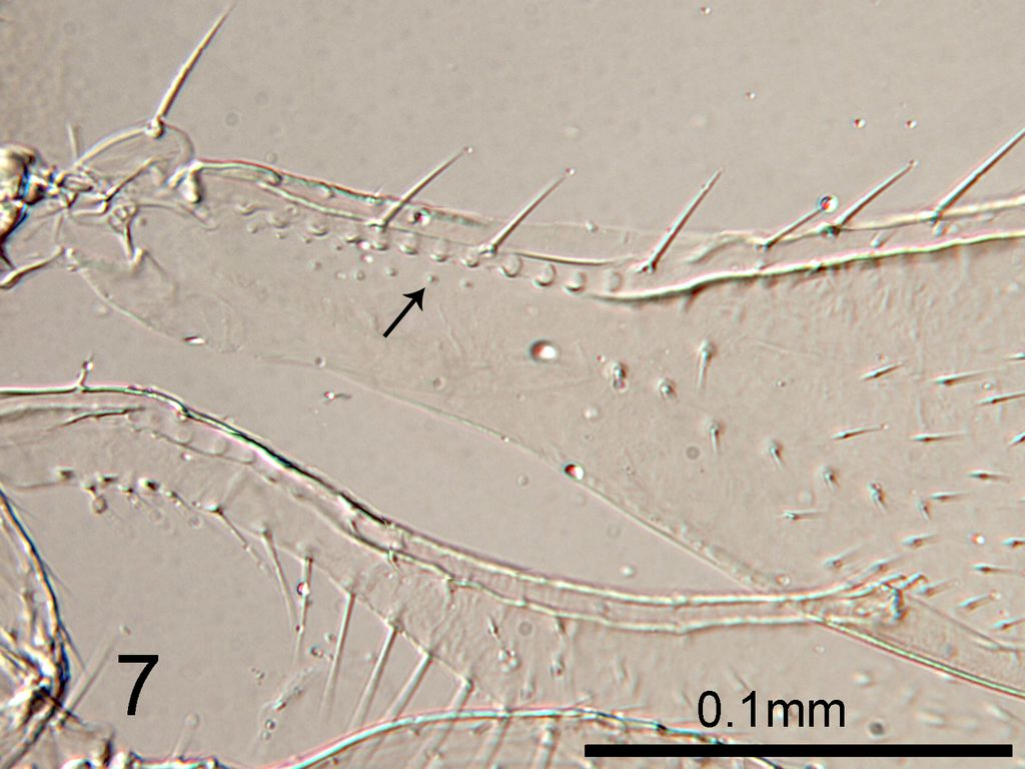
*Cales motterni*: detail of basal fore wing showing campaniform sensilla (arrowed)

**Figure 8.**
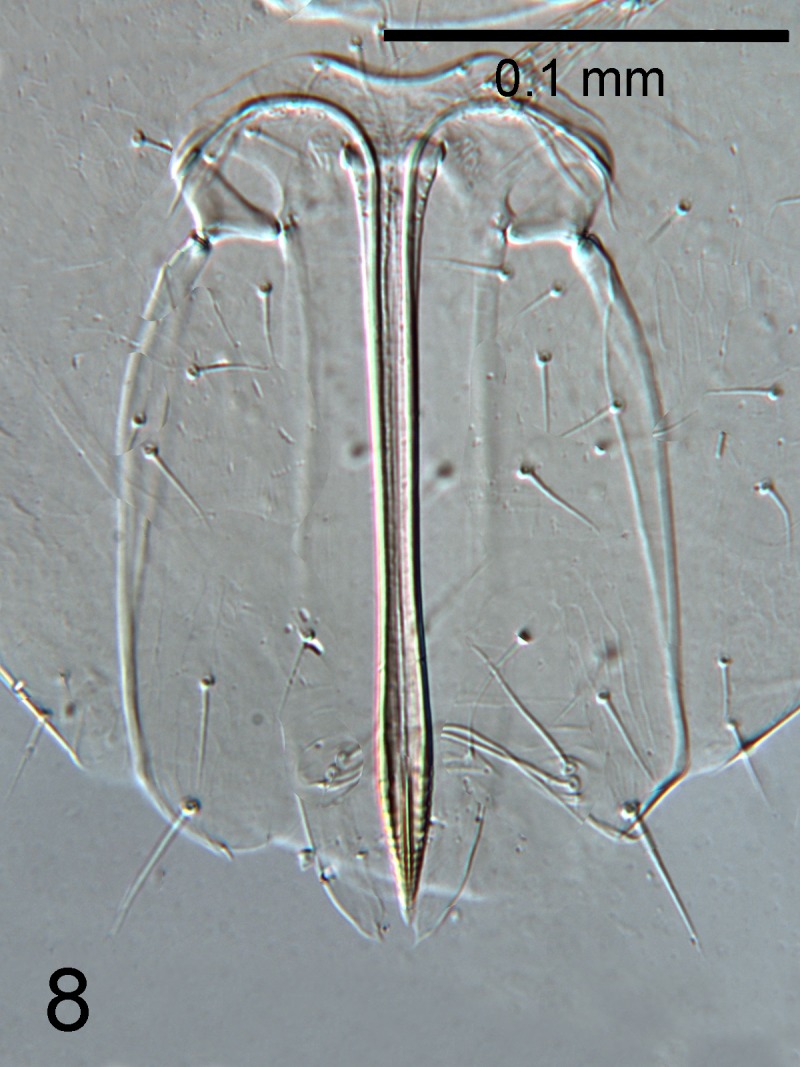
*Cales motterni*: ​ovipositor

Head with transverse sculpture, face ventral to antennae with scattered slender setae Fig. 1. Inter-antennal protuberance present (Fig. 2). Maxillary palp one-segmented. Antenna (Fig. 3) with radicle short, 1.1× as long as wide. Scape 4.8× as long as wide, 7.9× as long as radicle and 2.5× as long as pedicel, flagellum with four ﬂagellom­eres; f1 and f2 combined length shorter than f3, f3 2.1× as long as wide, shorter in length than pedicel plus f1 and f2, and 0.4× as long as clava; f3 with at least one basiconic peg sensillum basally, clava with 5-6 multiporous plate sensilla (arrowed in Fig. 4), apparently fused to the clava along their lengths; mps 0.1× length of clava. Claval setae 0.1× as long as clava; clava with an apparent partial suture approximately 1/3 along its length from the base (arrowed in Fig. 3). Clava 3.5× as long as wide, obliquely truncate api­cally. Lateral lobe of mesoscutum (Fig. 5) with one seta; mid lobe with two pairs of setae and faint reticulate sculpture; scutellum with two pairs of setae. Fore tibial spur 0.7× length of basitarsus. Fore wing (Fig. 6) hyaline, with faint infuscation basally, 3.3× as long as broad; longest seta of posterior marginal fringe 0.5× width of wing; marginal vein with row of six long setae along anterior margin; discal setation relatively uniform. A single row of small campaniform sensilla on dorsal surface of basal cell, just posterior to submarginal vein (Fig. 7). Hind wing 7.0× as long as broad, posterior marginal fringe 1.2× width of wing; discal setation arranged in 2-3 rows. Ovipositor (Fig. 8) 3.6× as long as hind basitarsus.

##### Male

Unknown.

#### Diagnosis

*Cales motterni* **sp.n.** can be distinguished from other species in the genus by the following combination of characters: antennal clava with several multiporous plate sensilla attached throughout their lengths; each side lobe of mesoscutum with one seta; fore wing with setae rather evenly distributed; a single row of small campaniform sensilla on dorsal surface of basal cell, just posterior to submarginal vein.

#### Etymology

The species is named for Dr Jason Mottern, formerly of the University of California, Riverside, USA in recognition of his major contribution to our understanding of this unusual genus.

#### Distribution

TAIWAN: Xindian District, Wulai.

#### Biology

A primary endoparasitoid of *Bemisia pongamiae* (Hemiptera: Aleyrodidae). No parasitoids have been recorded to date from this host (Noyes 2015; Shih et al. 2008).

#### Taxon discussion

The single female paratype is identical in all respects to the holotype. *Cales motterni* is an unusual species in several ways. Morphologically, the clava shows vestiges of having developed from a 2-segmented condition. This, plus the assumed plesiomorphic state of the wing setation, suggests it may be the most morphologically basal species known in the genus.

*Cales motterni* **sp.n.** is the only species of the genus currently known from Taiwan.

## Identification Keys

### Key to *Cales* species in the *C. spenceri*- group

**Table:** 

1	Fore wing disc with setae arranged in three distinct rows (Fig. 9). Neotropics and introduced into North America, the Mediterranean, Africa and Atlantic Ocean islands.	*C. noacki* species-group
–	Fore wing disc evenly setose or at most with a suggestion of the setae forming rows (e.g. Fig. 6), but setae never in three distinct rows. Old World only – Australia, New Zealand, Taiwan.	2
2	Fore wing with longest posterior marginal seta 0.8× width of wing. Mesoscutum with posterior setae long, more than one-third length of seta extending beyond transscutal articulation when directed posteriorly. Australia.	*C. spenceri* Girault
–	Fore wing with longest posterior marginal seta 0.5–0.6× width of wing (Fig. 6). Mesoscutum with posterior seta short, less than one-third length of seta extending beyond transscutal articulation when directed posteriorly. Australia, New Zealand, Taiwan.	3
3	Fore wing basal cell without campaniform sensilla, or sensilla present only as faint vestiges in proximal area posterior to submarginal vein. Fore wing with distinct infuscation posterior to submarginal and marginal veins. New Zealand.	*C. berryi* Mottern & Heraty
–	Fore wing with one or two rows of small campaniform sensilla on dorsal surface of basal cell, just posterior to submarginal vein (arrowed, Fig. 7). Fore wing hyaline or with faint infuscation. Australia, Taiwan.	4
4	Antenna with F3 elongate, about twice as long as wide; Clava with multiporous plate sensilla (arrowed, Fig. 4) apparently fused to clava along their lengths. Side lobes of mesoscutum each with 1 seta. Taiwan.	*C. motterni* Polaszek, Shih & Ward **sp. n.**
–	Antenna with F3 transverse, a little wider than long; Clava with multiporous plate sensilla (Fig. 10) clearly separate from clava along their lengths, attached only basally. Side lobes of mesoscutum each with 2 setae. Australia.	*C. orchamoplati* Viggiani & Carver

Adapted from Mottern et al. 2010.

## Supplementary Material

XML Treatment for Cales motterni

## Figures and Tables

**Figure 9.**
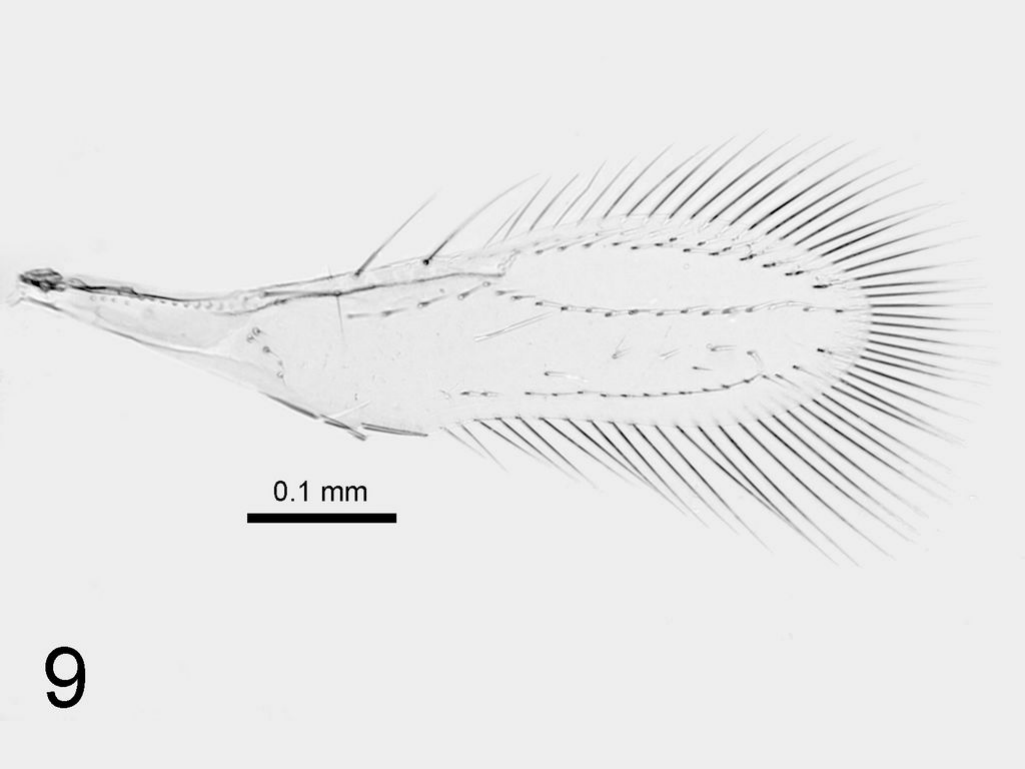
*Cales noacki*: fore wing

**Figure 10.**
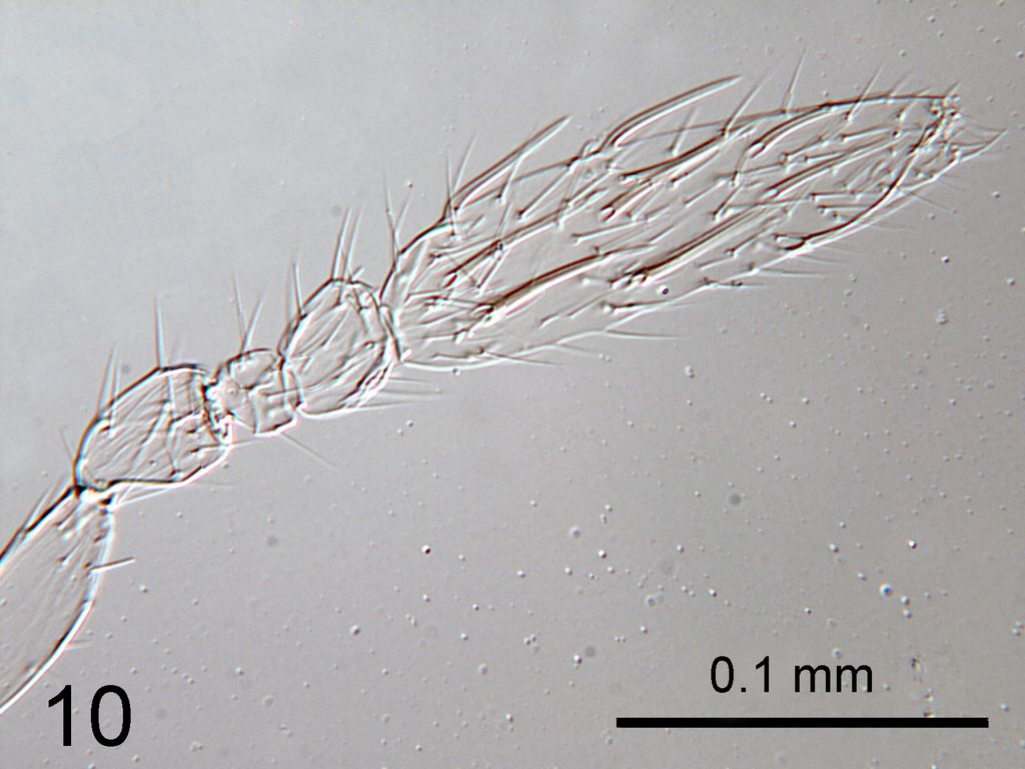
*Cales orchamoplati*: female antenna
